# A practical depth-of-interaction PET/MR detector with dichotomous-orthogonal-symmetry decoding

**DOI:** 10.1186/2197-7364-2-S1-A12

**Published:** 2015-05-18

**Authors:** Yuxuan Zhang, Hossain Baghaei, Han Yan, Wai-Hoi Wong

**Affiliations:** University of Texas MD Anderson Cancer Center, Houston, Texas USA

Conventional dual-end depth-of-interaction (DOI) PET detector readout requires two 2D SiPM arrays; with top and bottom SiPM reading the same pixel, there is information redundancy. We proposed a dichotomous-orthogonal-symmetric (DOS) dual-end DOI readout to eliminate this redundancy to significantly reduce SiPM usage, electronic channels, and heat load. Reflecting films are used within the scintillator array to channel light exiting the top along the X-direction, while light exiting the bottom is channeled along the orthogonal Y-direction. Despite the unidirectional channeling on each end, the top readout can provide X-Y information using two 1-D SiPM arrays; similarly, the bottom readout also provides X-Y information with two 1-D SiPM arrays. Thus four 1-D SiPM arrays (4xN) are used to decode XYZ to replace two 2D SiPM arrays (2NxN); SiPM usage is reduced from 2N**2 to 4N. Monte Carlo simulations (GATE) were carried out to study the XY decoding accuracy, energy resolution, and DOI resolution. Coupling the DOS-DOI design with a channel-decoding scheme, an array of 15x15 LSO (2.4x2.4x20 mm pixels) can be decoded by 18 SiPMs (2 rows of nine 3x3mm SiPM) on top and 18 SiPMs at bottom, thus achieving a 10X reduction in SiPM usage, electronic channels and heat load. For BGO detectors, an 8x8 array (2.4x2.4x20 mm pixels) can be achieved with 6.4X reduction. Simulations show 5-6mm DOI resolution, 0.45-0.96mm XY decoding blurring, 20-24% energy resolution. This study shows the feasibility of the DOS-DOI design. Even comparing to non-DOI detectors, there is a 5X/3X SiPM reduction for LSO/BGO. The proposed detector may yield practical ultrahigh-resolution PET/MR systems with depth-of-interaction with a production cost below current non-DOI systems.

